# Microbial upgrading of acetate into 2,3-butanediol and acetoin by *E. coli* W

**DOI:** 10.1186/s13068-020-01816-7

**Published:** 2020-10-22

**Authors:** Katharina Novak, Regina Kutscha, Stefan Pflügl

**Affiliations:** Research Area Biochemical Engineering, Environmental and Bioscience Engineering, Institute for Chemical, Technische Universität Wien, Gumpendorfer Straße 1a, 1060 Vienna, Austria

**Keywords:** Acetate utilization, Chemically defined medium, Aspartate, Acetate toxicity, *E. coli* W, Alternative raw materials, NADH availability, Amino acid and vitamin supplementation, Yeast extract

## Abstract

**Background:**

Acetate is an abundant carbon source and its use as an alternative feedstock has great potential for the production of fuel and platform chemicals. Acetoin and 2,3-butanediol represent two of these potential platform chemicals.

**Results:**

The aim of this study was to produce 2,3-butanediol and acetoin from acetate in *Escherichia coli* W. The key strategies to achieve this goal were: strain engineering, in detail the deletion of mixed-acid fermentation pathways *E. coli* W Δ*ldhA* Δ*adhE* Δ*pta* Δ*frdA* 445_Ediss and the development of a new defined medium containing five amino acids and seven vitamins. Stepwise reduction of the media additives further revealed that diol production from acetate is mediated by the availability of aspartate. Other amino acids or TCA cycle intermediates did not enable growth on acetate. Cultivation under controlled conditions in batch and pulsed fed-batch experiments showed that aspartate was consumed before acetate, indicating that co-utilization is not a prerequisite for diol production. The addition of aspartate gave cultures a start-kick and was not required for feeding. Pulsed fed-batches resulted in the production of 1.43 g l^−1^ from aspartate and acetate and 1.16 g l^−1^ diols (2,3-butanediol and acetoin) from acetate alone. The yield reached 0.09 g diols per *g* acetate, which accounts for 26% of the theoretical maximum.

**Conclusion:**

This study for the first time showed acetoin and 2,3-butanediol production from acetate as well as the use of chemically defined medium for product formation from acetate in *E. coli*. Hereby, we provide a solid base for process intensification and the investigation of other potential products.

## Background

**Fig. 1:**
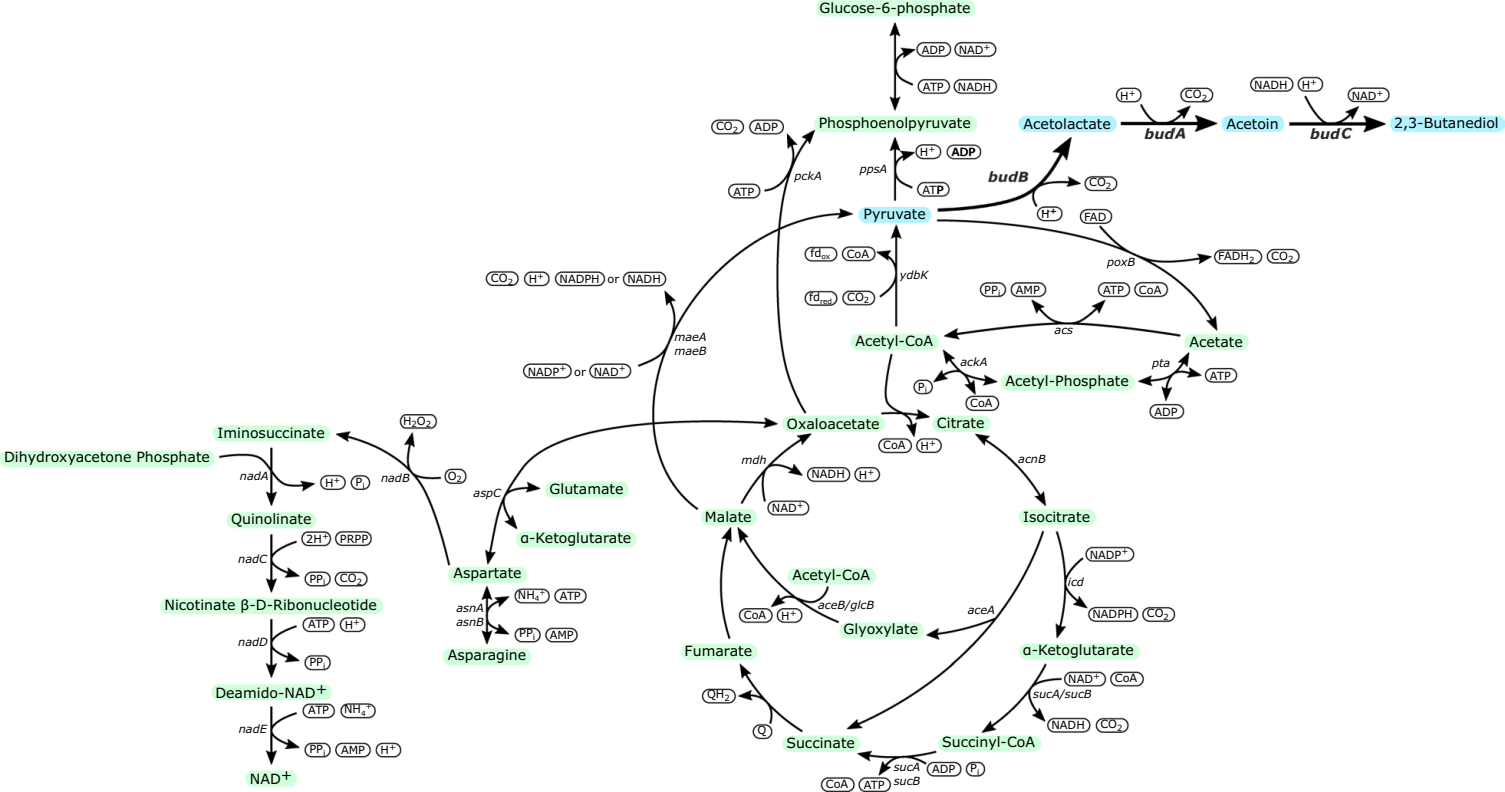
Metabolic network of *Escherichia coli* for 2,3-butanediol production from acetate. The figure includes the production pathway, acetate uptake, the TCA cycle, gluconeogenesis and the NAD^+^ de novo biosynthesis

Global rising energy demand, uncertainty about crude oil availability and concerns about climate change have recently increased the interest in renewable energy sources and microbial fuel and chemical production [1]. One promising platform chemical is 2,3-butanediol which can be used as food additive, anti-freezing agent [2] or as a precursor for butanone formation [3]. Similarly, acetoin is used as a flavor enhancer [4] or chemical building block [5]. Although 2,3-butanediol can be produced naturally by, e.g., *Klebsiella oxytoca* or *Enterobacter cloacae*, some hosts are pathogenic and require complex and expensive media additives [6]. Therefore, 2,3-butanediol production has recently been developed in *Escherichia coli* [6–8]. Since *E. coli* is not a natural producer, a heterologous pathway consisting of three genes has to be overexpressed: acetolactate synthase, acetolactate decarboxylase and butanediol dehydrogenase (Fig. 1) [9]. So far, 2,3-butanediol has been produced from glucose [7, 8, 10] and alternative sugar sources such as sugar beet molasses [6].

Acetate is a promising alternative raw material, since it can be derived from a variety of cost-effective resources such as food waste and lignocellulosic hydrolysates [11]. Moreover, acetate can also be produced in gas fermentations relying on C1 compounds which enables production in a cascaded process with two steps [12]. A broad spectrum of chemicals has already been produced from acetate such as polyhydroxyalkanoates [13], 3-hydroxypropionic acid [14] and succinate [15], and also alcohols like ethanol [16], isopropanol [17] and isobutanol [18]. So far, the vast majority of products that have been produced from acetate are derived from acetyl-CoA. The metabolic intermediate from where a product pathway branches plays an important role in microbial chemical production, as it influences the maximal theoretical yield that can be achieved. Three carbon compounds such as isopropanol, acetone and 3-hydroxypropionic acid, which are derived from acetyl-CoA or C3 products from the TCA cycle, like succinate, display a theoretical yield of 0.5 mol mol^−1^. In contrast, the theoretical yield of C4 alcohols produced from pyruvate like isobutanol and 2,3-butanediol is reduced to 0.25 mol mol^−1^ at the expense of higher CO_2_ emissions.

Acetate uptake in *E. coli* is possible via two routes: the high-affinity, irreversible acetyl-CoA synthetase (*acs*) or the low-affinity, reversible acetate kinase–phosphate acetyl transferase (*ackA*–*pta*) system [19]. Overexpression of either of these pathways has been shown to increase acetate uptake and product formation [14, 17, 20, 21]. Other strategies that resulted in increased product formation were the deletion of *icdA*, which led to blocking of the TCA cycle and increased acetone synthesis [21] or deletion of *iclR*, which activated the glyoxylate shunt and improved 3-hydroxypropionic acid production in combination with *acs* overexpression [14]. Generally, the glyoxylate shunt is highly active when acetate is used as sole carbon source, whereas the TCA cycle is down-regulated [19].

The main route for gluconeogenesis during growth on acetate is the formation of phosphoenolpyruvate (PEP) from oxaloacetate via phosphoenolpyruvate carboxykinase (*pckA*) or alternatively from malate via the malic enzymes and phosphoenolpyruvate synthase (*ppsA*) (Fig. 1) [19, 22]. A combined overexpression of *pckA*, the malic enzymes and *acs* showed improved isobutanol production from acetate [18].

Product formation from acetate has so far only been achieved by the addition of complex media components such as yeast extract. However, there are some major drawbacks and limitations related to the use of complex media additives [23]. It was recently shown that the availability of casamino acids led to differences in cellular metabolism, especially acetate metabolism in glucose-grown cultures [24]. Complex media additives are usually also cost-intensive and media cost was shown to be one of the core expenses in bioreactor cultivations [25, 26]. Media cost was mainly influenced by the feedstock price for peptones [25], which suggests that the omittance of complex media additives is beneficial for industrial applications. Additionally, Yang et al. [17] reported that these components can disguise the true product yield, since isopropanol production reached 112% of the maximal theoretical yield. Concluding, to maintain reproducibility, discover true physiological effects and reduce media costs, research should focus on the use of chemically defined medium for the investigation of acetate utilization.

Peptides and amino acids represent a major part of available substrates in yeast extract. Regarding acetate, different amino acids are known to protect cells against acid stress in the form of low pH. While the first acid resistance (AR) system is repressed in the presence of glucose, the activity of the other three systems is dependent on the availability of arginine (AR2), glutamate (AR3) or lysine (AR4) [27, 28]. Acetate has also been shown to inhibit the biosynthesis of methionine [29]. Apart from the toxicity of acetate, the low availability of NADH during acetate assimilation is not only a challenge in terms of energy conservation, but also for efficient production of reduced metabolites such as alcohols [30]. Aspartate is a precursor of the de novo NADH biosynthesis (Fig. 1) and its addition increased product formation in *Clostridium acetobutylicum* [31]. Carrying an amino group, amino acids can also have a stabilizing effect on the intracellular pH [32, 33]. Conclusively, amino acids play a major role during the assimilation of acetate.

The goal of this study was the production of 2,3-butanediol from acetate. To reach this goal, we investigated the influence of the strain background on product formation and developed a chemically defined medium containing amino acids and vitamins which enabled diol production from acetate. The stepwise reduction of media additives revealed that the addition of aspartate as sole amino acid is sufficient to trigger diol production from acetate. Moreover, using pulsed fed-batches we could prove that aspartate addition was only necessary in the batch phase, while acetate could be used as a sole carbon source for diol production during the feeding period.

## Results

### No production from acetate on complex medium containing yeast extract

In a previous study, 2,3-butandediol production from glucose was successfully established on chemically defined medium [6]. The two most promising strains, *Escherichia coli* W 445_Ediss (*W*) and *E. coli* *W* Δ*ldhA* Δ*adhE* Δ*pta* Δ*frdA* 445_Ediss (Δ4) were therefore chosen to investigate product formation from acetate. While *E. coli* *W* was able to utilize acetate in the original defined medium, *E. coli* *W* Δ4 was unable to grow. Both strains, however, grew well on the same medium when yeast extract was added. While *Escherichia coli* W only produced low amounts of 2,3-butanediol, product formation was 3.4-fold higher in *E. coli* *W* Δ4 (Additional file 1: Table S1). Given that aerobic conditions were used for this experiment, we did not expect that deleting mixed-acid fermentation pathways significantly influences 2,3-butanediol and acetoin formation from acetate. Conclusively, 2,3-butanediol and acetoin can be produced in *E. coli* W Δ4 on complex medium containing yeast extract.

**Fig. 2:**
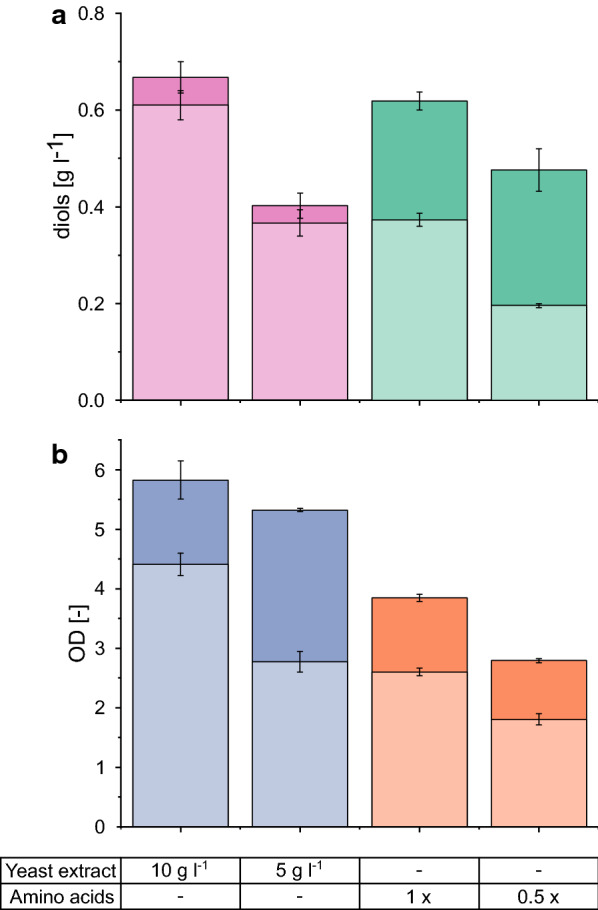
Comparison diol production (**a**) and growth (**b**) on complex and defined medium. *E. coli* W *ΔldhA ΔadhE Δpta ΔfrdA* 445_Ediss is compared in shake flasks with 10 g l^−1^ yeast extract or in defined medium (1- or 0.5-fold amino acid concentration and onefold vitamin concentrations). Light bars represent growth and production without the addition of acetate and dark bars with acetate. Mean values and standard deviations were calculated from biological triplicates

To determine the amount of diols produced from acetate, we omitted acetate in one approach and increased the yeast extract concentration in another approach (Fig. 2). This comparison clearly revealed that 2,3-butanediol and acetoin are almost exclusively formed from yeast extract, and not from acetate.

Based on these results, we investigated whether product formation from acetate is possible by designing a chemically defined medium.

### Development of defined medium enables diol production from acetate

Since the original chemically defined medium did not allow for growth and 2,3-butanediol production on acetate, the medium was expanded by specific components. These media components were based on literature reports on mechanisms behind 2,3-butanediol production as well as acetate toxicity. To this end, we selected several compounds as media additives: (i) glutamate, as intracellular glutamate pools are decreased in the presence of acetate [34] and glutamate is responsible for the acid resistance 2 (AR2) system [28]; (ii) arginine, which mediates the AR3 system; (iii) lysine, which mediates the less efficient AR4 system [27]; (iv) methionine due to the inhibition of the methionine biosynthesis in the presence of acetate [29]; (v) aspartate and nicotinic acid, since they are precursors of the NADH biosynthesis and NADH availability is low during acetate assimilation [31], and (vi) thiamine, which is a cofactor of acetolactate synthase, the first enzyme in the 2,3-butanediol production pathway. Other vitamins were added as reported to be beneficial for the growth of *E. coli* [35]. Finally, a chemically defined medium containing 5 different amino acids and 7 vitamins was designed.

In contrast to the original defined medium, the newly designed medium enabled growth and diol production from acetate. Diol formation was compared in experiments with and without the addition of acetate, which enabled us to quantify the amount of product formed from acetate. Figure 2 shows the production of 0.25 g l^−1^ diols from 5 g l^−1^ acetate. In other words, only the use of the newly designed defined medium allowed for acetoin and 2,3-butanediol production from acetate.

In addition to the design of a defined medium, the deletion of by-product formation pathways in *E. coli* W Δ4 was key to enable diol production from acetate. One of those deletions, the knock-out of *pta* also concerns acetate utilization and might therefore influence product formation from acetate. Generally, *E. coli* takes up acetate via two routes: the high-affinity, irreversible acetyl-CoA synthetase (*acs*) or the low-affinity, reversible acetate kinase–phosphate acetyl transferase (*ackA*-*pta*) system [19]. Therefore, we investigated whether the absence of one of the uptake systems, *pta*, can influence growth and product formation during acetate uptake. To this end, diol formation of *E. coli* W Δ4 was compared to *E. coli* W *ΔldhA ΔadhE* (Δ2) in Table 1.

**Table 1:** Comparison of diol production (2,3-butanediol and acetoin) in strains with and without deletion of *pta*

Strain	*E. coli* W Δ4	*E. coli* W Δ2
Compared experiments	1 AA ± 5 Ace	1 AA ± 5 Ace
Total diols [g l^−1^]	0.62 ± 0.03	0.65 ± 0.01
Diols from medium [g l^−1^]	0.37 ± 0.01	0.41 ± 0.01
Diols from acetate [g l^−1^]	0.25 ± 0.04	0.23 ± 0.02
*Y* [g diols g^−1^ acetate]	0.053 ± 0.009	0.045 ± 0.005

*E. coli* W Δ*ldhA* Δ*adhE* Δ*pta* Δ*frdA* 445_Ediss (Δ4) is compared to *E. coli* W *ΔldhA ΔadhE* 445_Ediss (Δ2) in shake flasks with defined medium and the onefold amino acid concentration and the onefold vitamin concentrations. Means and standard deviations were calculated from biological triplicates

Product formation in *E. coli* *W* Δ4 is comparable to *E. coli* *W* Δ2, which indicates that the deletion of *pta* does not influence the production of 2,3-butanediol. Therefore, all further experiments were carried out with *E. coli* W Δ4.

### Can the media additives be reduced?

**Fig. 3:**
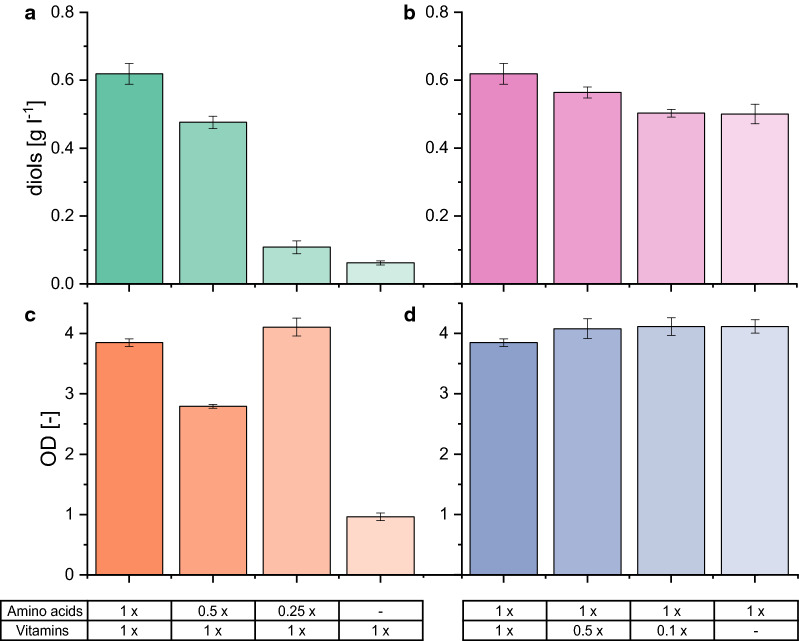
Effect of amino acid (**a, c**) and vitamin reduction (**b, d**) on diol production (**a, b**) and growth (**c, d**). *E. coli* W *ΔldhA ΔadhE Δpta ΔfrdA* 445_Ediss was used for cultivations in shake flasks. Mean values and standard deviations were calculated from biological triplicates

To better understand the mechanisms behind acetate uptake for diol production, our goal was to investigate which of the components in the designed medium are responsible for the observed effect and thereby essential. Therefore, we reduced or omitted the additives in a stepwise manner (Fig. 3). The reduction of the amino acid mix by 50% did not show an effect on growth and diol production, but reduction to 25% of the original composition resulted in decelerated growth and loss of diol production. Growth was not possible on medium without the addition of amino acids (Fig. 3a). In contrast, the reduction of the vitamin concentration only slightly decreased the final product titers and completely omitting vitamins still enabled the production at 83% of the diol concentration with the onefold vitamin concentration.

### Asparagine and aspartate trigger diol production from acetate

**Fig. 4:**
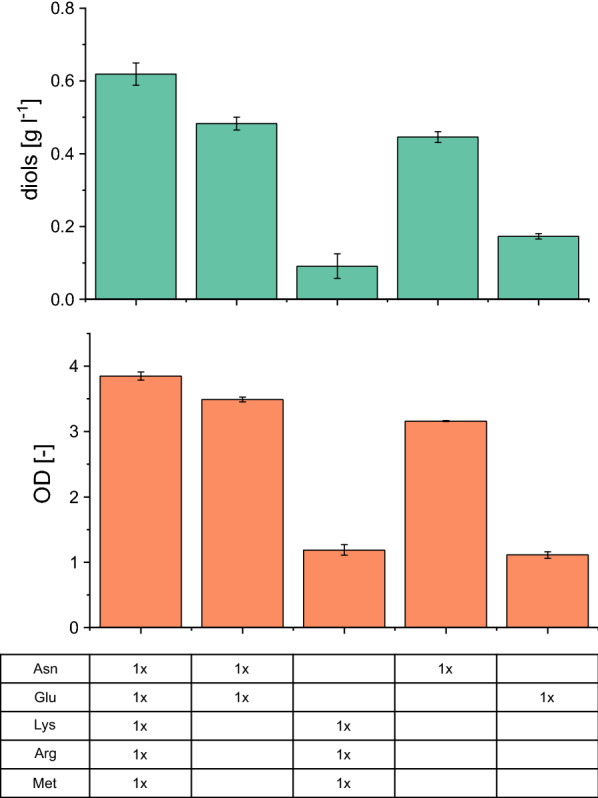
Effect of addition of single amino acids and groups of amino acids on diol production (**a**) and growth (**b**). *E. coli* W *ΔldhA ΔadhE Δpta ΔfrdA* 445_Ediss was used for cultivations on defined medium additionally containing the onefold vitamin concentration in shake flasks. Mean values and standard deviations were calculated from biological triplicates

To further investigate the influence of single components of the designed medium on diol formation from acetate, we systematically removed several media additives from the chemically defined medium. Therefore, the five amino acids were grouped: asparagine and glutamate as the main amino acids present in yeast extract were compared to the mixture of arginine, lysine, and methionine (Fig. 4). While the overall concentration of amino acids was similar in both approaches, only the asparagine–glutamine group resulted in product formation comparable to the defined medium containing all amino acids. By adding asparagine or glutamate at the onefold concentration as sole amino acids, we could show that the presence of asparagine and glutamate as sole amino acid resulted in diol concentrations of 0.44 g l^−1^ and 0.17 g l^−1^, respectively. Supplementing arginine, lysine, and methionine individually at higher concentrations (corresponding to the mass of asparagine), enabled growth on acetate but did not trigger diol production (Additional file 1: Table S2).

Diol formation from acetate was quantified similarly to previous experiments by comparing media with and without acetate. Additionally, we tested whether diol formation from acetate can also be achieved by the addition of aspartate instead of asparagine. To quantitatively verify the amount of diol produced from acetate, different amounts of amino acids were used in experiments with a constant acetate concentration (Table 2).

**Table 2:** Comparison of diol production (2,3-butanediol and acetoin) in *E. coli* W Δ4 on different defined media

Experiments	1 AA ± 5 Ace	0.5 AA ± 5 Ace	2 Asn ± 5 Ace	1 Asn ± 5 Ace	1 Asp ± 5 Ace
Total diols [g l^−1^]	0.62 ± 0.03	0.48 ± 0.02	0.42 ± 0.02	0.35 ± 0.01	0.35 ± 0.01
Diols from medium [g l^−1^]	0.37 ± 0.01	0.20 ± 0.01	0.19 ± 0.01	0.08 ± 0.01	0.10 ± 0.01
Diols from acetate [g l^−1^]	0.25 ± 0.04	0.28 ± 0.02	0.24 ± 0.02	0.27 ± 0.01	0.26 ± 0.02
*Y* [g diols g^−1^ acetate]	0.053 ± 0.009	0.062 ± 0.005	0.049 ± 0.005	0.052 ± 0.002	0.052 ± 0.004

*E. coli* W Δ*ldhA* Δ*adhE* Δ*pta* Δ*frdA* 445_Ediss was used for cultivation. Media containing the five amino acid mixture (AA) is compared to medium containing 0.88 g l^−1^ (onefold) or 1.76 g l^−1^ (twofold) asparagine (Asn) or aspartate (Asp) and the onefold vitamin concentration. The diol concentration from acetate is calculated by the subtraction of the concentration attributed to the amino acid(s). Mean values and standard deviations were calculated from biological triplicates

When asparagine or aspartate are used instead of the amino acid mixture, product formation from acetate was equal, whereas the overall product formation from the medium is decreased. Equal yields in all approaches indicate that all other amino acids except asparagine simply increased the media background, but did not significantly improve product formation from acetate. The use of aspartate instead of asparagine led to comparable diol yields and is therefore equally suitable as a media additive. Reduction of the aspartate concentration led to decelerated acetate uptake and growth. A decrease to 50% of the initial concentration drastically reduced growth and 2,3-butanediol production (Table 3).

**Table 3:** Diol production in medium containing different amounts of aspartate in *E. coli* W Δ4

Aspartate reduction	100% (8 mol%)	50% (4 mol%)	25% (2 mol%)	10% (0.8 mol%)
OD [−]	2.92 ± 0.08	0.84 ± 0.03	0.66 ± 0.02	0.62 ± 0.01
Total diols [g l^−1^]	0.35 ± 0.01	0.12 ± 0.01	0.06 ± 0.01	0.03 ± 0.01

*E. coli* W Δ*ldhA* Δ*adhE* Δ*pta* Δ*frdA* 445_Ediss was used for cultivation. Media contained 5 g l^−1^ acetate and the indicated percentage of 0.88 g l^−1^ aspartate. The ratio of aspartate to acetate is given in % of moles. Cultures were inoculated at OD_600_ = 0.5. Mean values and standard deviations were calculated from biological triplicates

### Elucidating the mechanism behind the aspartate/asparagine effect

To further investigate how the addition of asparagine or aspartate enables product formation from acetate, we hypothesized about possible physiological mechanisms behind this effect. Since the addition of other amino acids did not result in product formation from acetate, asparagine or aspartate might act as a precursor for other important metabolic processes. It is possible that aspartate boosts gluconeogenesis by conversion to oxaloacetate and phosphoenolpyruvate (Fig. 1). Alternatively, asparagine or aspartate could support the flux through the citrate or glyoxylate cycle. According to these hypotheses, other TCA cycle intermediates should also promote growth and diol production from acetate. Therefore, aspartate in the medium was replaced by oxaloacetate, succinate, and malate (Table 4).

**Table 4:** Comparison of diol production (2,3-butanediol and acetoin) in *E. coli* W Δ4 from different TCA intermediates

Compared experiments	1 Mal ± 5 Ace	1 Suc ± 5 Ace	1 OAA ± 5 Ace	1 Asn ± 5 Ace
OD [−]	0.95 ± 0.04	0.99 ± 0.06	0.53 ± 0.03	3.16 ± 0.01
Total diols [g l^−1^]	0.12 ± 0.01	0.14 ± 0.01	0.02 ± 0.01	0.35 ± 0.01
Diols from medium [g l^−1^]	0.05 ± 0.01	0.07 ± 0.01	0.03 ± 0.01	0.08 ± 0.01
Diols from acetate [g l^−1^]	0.06 ± 0.01	0.07 ± 0.02	− 0.01 ± 0.01	0.27 ± 0.01

*E. coli* W Δ*ldhA* Δ*adhE* Δ*pta* Δ*frdA* 445_Ediss was used for cultivation. Media containing 0.88 g l^−1^ malate (Mal), succinate (Suc), oxaloacetate (OAA) and asparagine (Asn) with the onefold vitamin concentration were compared. The diol concentration from acetate is calculated by the difference of experiments with and without acetate or by the subtraction of the concentration attributed to the amino acid(s). Mean values and standard deviations were calculated from biological triplicates

None of these alternative media supplements supported growth and diol production from acetate. It is likely that the mechanism behind aspartate triggering diol production is different. Therefore, we speculated that aspartate mediates a form of acid resistance. This hypothesis is supported by the observation that a decrease of the initial acetate concentration from 5 g l^−1^ to 2 g l^−1^ enabled growth and production without the addition of amino acids or vitamins. However, cultivations at low substrate concentrations are hardly feasible due to the low product titer which additionally complicates quantification.

### Co-utilization or start-kick?

After narrowing down the possible mechanisms mediated by aspartate, it was important to find out how aspartate uptake influences acetate uptake and diol production in a time-resolved manner. To this end, the kinetics of aspartate and acetate uptake as well as product formation were studied in bioreactor cultivations, which also reduced distortions due to pH increase caused by acetate uptake from the medium in shake flasks.

Generally, acetate-grown cultures displayed a high degree of variation between individual cultivations. These variations could be reduced but not completely prevented by: (i) the adaptation of cells to defined medium in the preculture and (ii) the transfer of the preculture to the reactor in the exponential phase. These measures resulted in reproducible results in terms of product concentrations and yields.

The batch cultivations as well as all further cultivations were carried out on defined medium containing 5 g l^−1^ acetate, 0.88 g l^−1^ (8 mol%) aspartate and the onefold concentration of vitamins. Although experiments in shake flasks did not require vitamin addition for 2,3-butanediol and acetoin formation, we observed that omitting vitamins in bioreactor cultivations led to a 50% decreased product yield and only acetoin rather than 2,3-butanediol production (data not shown). Therefore, vitamins were added at the onefold concentration in all further experiments.

**Fig. 5:**
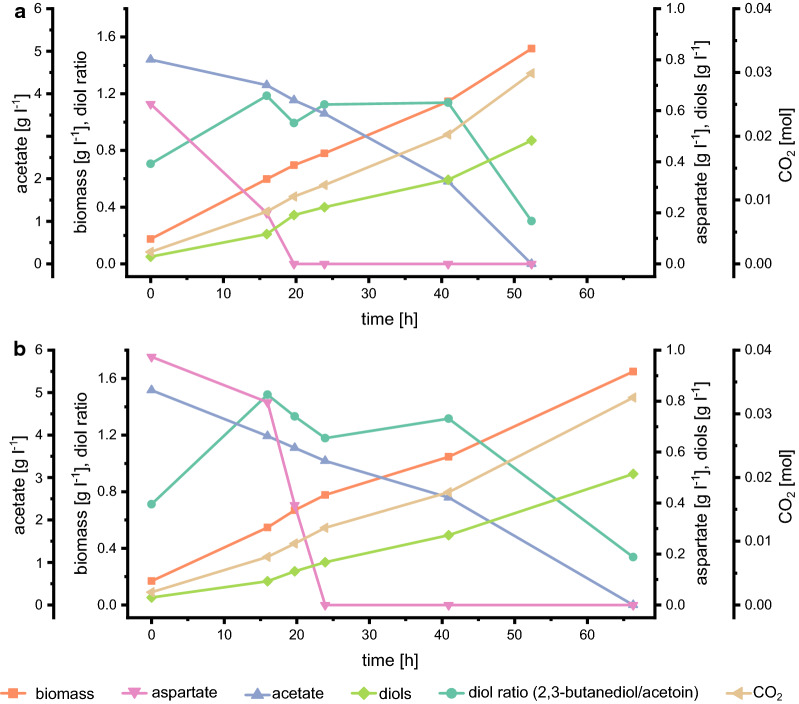
Results from duplicate batch experiments on defined medium containing acetate and aspartate. *E. coli* W *ΔldhA ΔadhE Δpta ΔfrdA* 445_Ediss was used for cultivations on defined medium containing 5 g l^−1^ acetate and the onefold aspartate and vitamin concentration

Figure 5 shows that growth and diol production from acetate and aspartate is possible in batch experiments. Growth occurred in two phases: in the first one, acetate was partially, and aspartate completely consumed while in the second phase the remaining acetate was utilized. This diauxic growth pattern indicates that aspartate is only needed to give a start-kick for growth and that co-utilization is not required for product formation from acetate. Although we maintained reproducibility by adapting the preculture, cultures still varied in substrate uptake as well as production rates.

Product yields were higher than in shake-flask experiments and reached 26% of the theoretical maximum in the acetate phase (Table 5).

**Table 5:** Process performance parameters of *E. coli* W Δ4 in batches with aspartate and acetate

**Parameter**	Aspartate phase	Acetate phase	Total
*Y*_diols/Ace_ [Cmol Cmol^−1^]	0.18 ± 0.09	0.13 ± 0.03	0.14 ± 0.01
*Y*_diols/Ace_ [g g^−1^]	0.14 ± 0.07	0.09 ± 0.02	0.10 ± 0.01
*Y*_diols/S_ [Cmol Cmol^−1^]	0.11 ± 0.05	0.13 ± 0.03	0.12 ± 0.01
*Y*_*X/S*_ [Cmol Cmol^−1^]	0.36 ± 0.09	0.29 ± 0.05	0.31 ± 0.01
*Y*_CO2/S_ [Cmol Cmol^−1^]	0.71 ± 0.18	0.84 ± 0.11	0.78 ± 0.01
Carbon balance [%]	105 ± 9	112 ± 10	117 ± 1

*E. coli* W Δ*ldhA* Δ*adhE* Δ*pta* Δ*frdA* 445_Ediss was used for cultivation. Yields are either calculated per acetate consumed (*Y*_diols/Ace_) or per acetate and aspartate consumed (*Y*_diols/S_, *Y*_*X/S*_*, Y*_CO2/S,_ *X* = biomass, Ace = acetate, *S* = substrate (acetate + aspartate)). The medium contained the onefold aspartate (0.88 g l^−1^) and vitamin concentration. Mean values and standard deviations were calculated from biological duplicates. The theoretical yield is 0.5 Cmol diols per Cmol acetate

### Efficient diol production in pulsed fed-batches

**Fig. 6:**
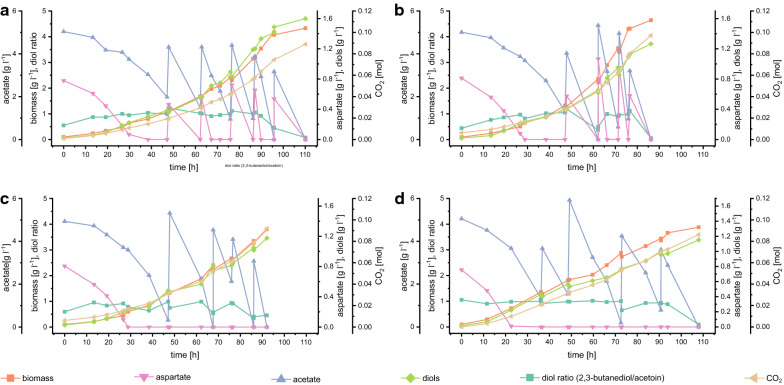
Results from duplicate fed-batch experiments on defined medium and pulses with acetate and aspartate (**a**, **b**) or acetate (**c**, **d**). *E. coli* W *ΔldhA ΔadhE Δpta ΔfrdA* 445_Ediss was used for cultivations. The batch media contained defined medium with 5 g l^−1^ acetate and the onefold aspartate and vitamin concentration. A mixture of acetate and aspartate (**a**, **b**) or acetate alone (**c**, **d**) was added in pulses to obtain an acetate concentration of 4 g l^−1^

Finally, we aimed to gain deeper insight into the mechanisms of the two-substrate system and to increase product titers and production rates. Therefore, we tested whether the addition of aspartate was necessary only during the batch or also in the feeding period of a pulsed fed-batch. To this end, pulses with a mixture of aspartate and acetate were compared to pulses where acetate was used as sole carbon source. To obtain comparability, all experiments were pulsed until they had consumed the same amount of acetate. It seems that aspartate was depleted before acetate during every pulse (Fig. 6). In all cultivations, a mixture of 2,3-butanediol and acetoin was produced, and the product spectrum shifted towards acetoin in later cultivation phases. Production of acetoin rather than 2,3-butanediol is probably caused by insufficient NADH supply during acetate utilization.

Pulsing aspartate in addition to acetate led to a 20% increase in the final titer (Table 6). Overall product yields (*Y*_diols/S_) were identical in approaches with and without aspartate. Since the absolute amount of acetate pulsed is equal in both experiments and they just differ in aspartate, which was added in one approach, it seems like the increased product titers are mainly caused by the additional carbon source in the form of aspartate. Figure 6 shows that aspartate is only needed for a start-kick in the first batch and that pulses with acetate as the sole carbon source are successfully used for diol production. Moreover, acetate uptake and diol production rates do not differ between experiments with only acetate or the two-substrate system, which indicates that the addition of aspartate does not improve acetate uptake and diol production. Similarly, the addition of aspartate does not increase biomass yields or specific uptake and production rates.

**Table 6:** Process performance parameters of *E. coli* W Δ4 in pulsed fed-batches with aspartate and acetate or solely acetate

	Acetate + aspartate pulses	Acetate pulses
**Titer, yields and carbon balance**		
Diols [g l^−1^]	1.43 ± 0.24	1.16 ± 0.02
*Y*_diols/Ace_ [g g^−1^]	0.075 ± 0.06	0.067 ± 0.02
*Y*_diols/Ace_ [Cmol Cmol^−1^]	0.103 ± 0.009	0.091 ± 0.003
*Y*_diols/S_ [Cmol Cmol^−1^]	0.087 ± 0.008	0.087 ± 0.003
*Y*_X/S_ [Cmol Cmol^−1^]	0.24 ± 0.01	0.24 ± 0.01
*Y*_CO2/S_ [Cmol Cmol^−1^]	0.64 ± 0.02	0.69 ± 0.01
Carbon balance [%]	110 ± 1	117 ± 1
**Volumetric and specific rates**		
*r*_Ace_ [g l^−1^ h^−1^]	0.21 ± 0.03	0.20 ± 0.03
*r*_diols_ [g l^−1^ h^−1^]	0.015 ± 0.001	0.013 ± 0.001
*q*_Ace_ [g g^−1^ h^−1^]	0.25 ± 0.01	0.24 ± 0.04
*q*_diols_ [g g^−1^ h^−1^]	0.018 ± 0.003	0.016 ± 0.002

*E. coli* W Δ*ldhA* Δ*adhE* Δ*pta* Δ*frdA* 445_Ediss was used for cultivation. The sum of 2,3-butanediol and acetoin is indicated as diols. Yields are either calculated per acetate consumed (*Y*_diols/Ace_) or per acetate and aspartate consumed (*Y*_diols/S_, *Y*_*X/S*_, *Y*_CO2/S_, *X* = biomass, Ace = acetate, *S* = substrate (acetate + aspartate)). Rates (volumetric rate r and specific rate q (per biomass)) calculated for the time course of the whole process. The media contained the onefold aspartate and vitamin concentration. Mean values and standard deviations were calculated from biological duplicates. The theoretical yield is 0.5 Cmol diols per Cmol acetate

Conclusively, aspartate addition is only necessary for a start-kick in the first batch phase and acetate can be used as the sole carbon source to produce 2,3-butanediol and acetoin during the feeding period without negatively affecting productivity or product yields.

### Low diol production in continuous culture

Continuous bioprocessing is a very promising tool to investigate physiological mechanisms and we sought to investigate diol production from acetate in substrate-limited cultures at steady-state conditions. Continuous cultivation is also an opportunity to increase productivity. To this end, the influence of co-feeding acetate and aspartate on product formation was evaluated in chemostat experiments.

Although both carbon sources were completely consumed, diol production decreased to 0.003 ± 0.002 g l^−1^ h^−1^. Co-utilization of acetate and aspartate resulted in the production of 0.009 ± 0.007 g diols per *g* substrate. This corresponds to only 15% and 11%, respectively, of what was reached in the pulsed fed-batches.

Consequently, continuous cultures under the conditions chosen are not suitable for production of 2,3-butanediol from acetate.

## Discussion

In this study, we showed that a knock-out strain lacking mixed-acid fermentation pathways can produce acetoin and 2,3-butanediol from acetate. Since a complex medium containing yeast extract did not allow for production from acetate, we designed a new chemically defined medium which enabled acetoin and 2,3-butanediol formation from acetate. By further reduction of this medium, we could reduce the additives to aspartate, which was only needed to give cultures a start-kick.

Microbial chemical production from acetate is an emerging field and commonly relies on the use of complex media additives [17, 18, 20, 21]. Therefore, it was surprising that the addition of yeast extract did not allow 2,3-butanediol and acetoin formation from acetate, but only from yeast extract. In another study investigating isobutanol production from acetate product formation in complex acetate medium was also compared to a control without acetate, and it was found that yeast extract accounted for about 50% of the total product formation with 40 mg l^−1^ isobutanol being produced from acetate alone [18]. Our results underline the importance of examining product formation from the “background medium” without the designated carbon source. Evaluating the “background production” is therefore especially important when low titers are to be expected, which would be the case for substrates displaying toxicity or a low-energy content such as acetate. Generally, the use of complex media comes with drawbacks and limitations, which have recently been intensively investigated and described [23]. For instance, the addition of amino acids resulted in differences of cellular metabolism, especially of acetate metabolism [24]. Although no data are available for acetate-grown cultures, changes in acetate metabolism are a possible explanation for the deviations between complex and defined medium observed in this study.

The fact that the deletion of mixed-acid fermentation pathways is relevant for product formation on acetate as the sole carbon source has not been reported before. A major focus in research on acetate utilization was laid on genetic engineering of the acetate assimilation system. It was previously shown that the overexpression of *acs* could reduce the lag phase during cultivations using acetate as sole carbon source and this indicates that acetate uptake can be improved at the expense of enhanced energy demand [36]. The great importance of *acs* for microbial production from acetate is also suggested by the comparison of two studies: while the overexpression of *pta–ackA* only increased acetone and isopropanol yields by 8 and 13%, respectively [17, 21], the production of 3-hydroxypropionic was improved by 75% by overexpressing *acs* [14]. With acetate as the sole carbon source, acetyl-CoA synthetase (*acs*) is the main route for acetate uptake, while *pta–ackA* is down-regulated [37]. This expression pattern probably accounts for enhanced effects by *acs* rather than *pta–ackA* overexpression. Similarly, the low expression of *pta* during acetate assimilation might be the reason that we did not find any differences in product formation between *E. coli* *W* Δ4 with *pta* knocked-out and *E. coli* *W* Δ2. We hypothesized that low activity of the reversible *pta–ackA* pathway can positively influence growth and product formation, since it prevents acetic acid cycling in the *acs–pta–ackA* node and avoids wasting of energy [38]. However, the activity of *pta–ackA* is important for providing acetyl-phosphate, which is essential for proper growth of *E. coli* [38]. In detail, acetyl-phosphate plays a role in degradation of misfolded proteins [39], phosphate and nitrogen assimilation [40, 41], survival during starvation [42] and gene expression [43]. It seems like acetyl-phosphate formation from acetate via *ackA* is sufficient to maintain all cellular functions, since growth and production did not differ between *E. coli* *W* Δ4 (*pta* deleted) and *E. coli* *W* Δ2. The activity of *acs* was also reported to depend on post-translational acetylation [44, 45] and its extent might vary within different media and process conditions. While the deletion of *pta* did not affect diol production, it seems that deletions of the by-product formation pathways for lactate and ethanol (*ldhA* and *adhE*) are important for product formation, since *E. coli* *W* Δ2 and Δ4 produced equally well, whereas *E. coli* *W* failed to produce diols from acetate. Comparing this study to recent literature [14, 15, 17, 18] shows that the major difference is the metabolic intermediate, from which the product is derived. While the precursor for other products such as acetone, isopropanol, 3-hydroxypropionic acid and phloroglucinol is acetyl-CoA [14, 17, 20, 21], 2,3-butanediol is derived from pyruvate. Therefore, to produce 2,3-butanediol from acetate, acetate must first be converted into pyruvate. To provide pyruvate, acetate is converted into acetyl-CoA, which is transformed to oxaloacetate or malate via the TCA cycle and from there PEP or pyruvate is formed. All factors that influence this conversion and thereby increase the intracellular pyruvate pool might be key for product formation. In this context, pyruvate availability is not only important for anabolic reactions, when cells are grown on acetate, but also for 2,3-butanediol formation.

The main routes for gluconeogenesis (*pckA* and malic enzymes + *ppsA*) branch from oxaloacetate or malate and are highly active during growth on acetate [37]. Because aspartate is a precursor for oxaloacetate, the addition of aspartate could enable growth and product formation by supporting gluconeogenesis via *pckA*. This hypothesis is in accordance with the observation that overexpression of *pckA* improved phloroglucinol synthesis [20]. In *C. glutamicum,* aspartate addition inhibited PEP carboxylase, which catalyzes the reverse reaction of *pckA* [46]. Combined overexpression of *acs*, *pckA* and the malic enzymes increased isobutanol production from acetate [47]. However, since the addition of oxaloacetate did not enable growth and product formation from acetate in our study, an influence of aspartate on gluconeogenesis via *pckA* is unlikely. Similarly, an influence of aspartate on the citric acid or glyoxylate cycle is questionable, since the addition of neither succinate nor malate triggered growth or product formation from acetate. An activation of the glyoxylate shunt was previously shown to improve acetate assimilation and biomass formation, but not 3-hydroxypropionic acid formation [14].

Alternatively, aspartate has been described to trigger acid resistance in different microorganisms and based on a variety of mechanisms. In *Yersinia pseudotuberculosis* aspartate increased acid resistance through expression of aspartase, which released NH_4_^+^ for the stabilization of the intracellular pH [33]. Aspartate also increased the NADH de novo biosynthesis and has been shown to increase product formation in *Clostridium acetobutylicum* [31]. Besides these two mechanisms, aspartate increased acetate formation in *Acetobacter pasteurianus* by reducing acid stress due to (i) enhanced glutathione production, which protects cells against low pH and stress; (ii) increased nucleic acid synthesis and DNA repair and (iii) improved membrane integrity due to increased fatty acid synthesis [32]. It is possible that one or several of the described mechanisms account for the effect that aspartate triggered diol production from acetate in this study. The fact that aspartate is a precursor for NADH synthesis is especially interesting in this context, because NADH availability is important for the conversion of acetoin to 2,3-butanediol. However, increased NAD^+^ biosynthesis mediated by aspartate availability does not necessarily mean that it is present as NADH. All experiments showed a mixture of 2,3-butanediol and acetoin in acetate-grown cultures. When vitamins were omitted, total diol production decreased, and the reaction shifted towards acetoin. The addition of vitamins to batch cultures might be important for diol formation, since thiamine is a cofactor for acetolactate synthase or since nicotinic acid is a precursor for NAD^+^ biosynthesis. Conclusively, these observations indicate insufficient NADH supply and possibly a critical redox status of the cell, which was at least partially circumvented by the addition of aspartate.

Acid resistance systems that are dependent on the availability of an amino acid have been reported to rely on glutamate, arginine and lysine, but not on aspartate [27, 28]. Therefore, it is surprising that the addition of these components did not enable growth on acetate. Similarly, the addition of methionine did not support growth, although its biosynthesis is known to be inhibited during growth on acetate [29].

Aspartate being consumed before acetate might suggest that aspartate simply gives cultures a start-kick by boosting biomass formation to a certain level. However, utilization of other substrates (succinate, glutamate, etc.) also increased initial biomass formation without leading to acetate utilization and/or product formation.

Pulsed fed-batches were successfully applied for efficient diol production. The production of 1.43 g l^−1^ diols is the highest titer obtained for any pyruvate-derived metabolite from acetate. Yang et al. [17] produced 1.5 g l^−1^ isopropanol from acetate via acetyl-CoA at a yield above the theoretical maximum (due to the addition of complex media additives). When products are derived from the TCA cycle, higher titers were achieved, i.e., 3.6 g l^−1^ itaconic acid and 7.5 g l^−1^ succinate were produced from acetate in *E. coli* [48, 49].

Low productivities in continuous cultures could suggest that co-utilization of aspartate and acetate might negatively affect diol production or that substrate limitation decreases product formation as reported before for isobutanol production from glucose [50]. However, how 2,3-butanediol production from acetate can be improved in continuous cultures requires further investigations.

The importance of this study lies in the use of chemically defined medium for microbial chemical production from acetate. Since the addition of aspartate is only needed for a start-kick during the first batch, the designed process is especially promising for further research focusing on industrial applications.

To further optimize the system, acetate uptake and production should be improved in combination with an enhancement of the product yield, e.g., by optimizing pyruvate availability. Better understanding of the mechanisms behind the importance of aspartate addition can provide targets for genetic engineering and to completely avoid media additives in the future. Those mechanisms could, e.g., be elucidated in transcriptome and metabolome studies. Overexpression of the NADH biosynthesis pathway could potentially bypass the addition of aspartate and improve product formation on acetate. Future research might also shed light on the effect of the deletions in *E. coli* *W* Δ2 and Δ4 on product formation and acetate toxicity and provide information for the development of a continuous production process.

## Conclusion

In this study, we showed for the first time (i) 2,3-butanediol and acetoin production from acetate; (ii) product formation from acetate in *E. coli* on chemically defined medium and (iii) triggering of diol production by addition of aspartate. Yeast extract was demonstrated to be an unsuitable media additive for 2,3-butanediol and acetoin production from acetate. Therefore, a chemically defined medium was designed and reducing the media additives for the first time showed that the addition of aspartate alone enabled product formation from acetate. Furthermore, aspartate is only needed to give the culture a start-kick and acetate can subsequently be used as the sole carbon source for product formation.

Concluding, we could create a picture of conditions influencing production performance of a microbial system utilizing acetate. For instance, we observed that reproducibility could be improved by adapting precultures in defined medium and inoculating during the exponential phase. Therefore, the combined outcome of this study provides a sound basis for further optimization and investigation of the production of 2,3-butanediol and other platform chemicals from acetate.

## Methods

### Bacterial strains and media

*E. coli* *W* (DSM 1116 = ATCC 9637 = NCIMB 8666) from DSMZ (Braunschweig, Germany), *E. coli* *W* Δ*ldhA* Δ*adhE* Δ*pta* Δ*frdA* and *E. coli* W Δ*ldhA* Δ*adhE* (kind gifts of Prof. Michael Sauer, BOKU, Vienna, Austria) were used for cultivations.

Lysogeny broth (LB) containing 10 g l^−1^ soy peptone, 5 g l^−1^ yeast extract and 10 g l^−1^ sodium chloride was used for all precultures in the shake-flask experiments. 15 g l^−1^ agar was added to LB medium for cultivation on plates.

All chemicals were purchased from Roth (Carl Roth GmbH + Co. KG, Karlsruhe, Germany) if not stated otherwise. The basic medium for all experiments is chemically defined medium adapted from Riesenberg et al. [51], containing 13.3 g l^−1^ KH_2_PO_4_, 4.0 g l^−1^ (NH_4_)_2_HPO_4_, 1.7 g l^−1^ citric acid (autoclaved) 1.2 g l^−1^ MgSO_4_ * 7 H_2_O, 0.10 g l^−1^ Fe(III)citrate, 0.0084 g l^−1^ EDTA, 0.013 g l^−1^ Zn(CH_3_COO)_2_ * 2 H_2_O, 0.0025 g l^−1^ CoCl_2_ * 6 H_2_O (Merck KGaA, Darmstadt, Germany), 0.015 g l ^−1^ MnCl_2_ * 4 H_2_O, 0.0012 g l^−1^ CuCl_2_ * 2 H_2_O, 0.0030 g l^−1^ H_3_BO_3_, 0.0025 g l^−1^ Na_2_MoO_4_ * 2 H_2_O (sterile filtered). Acetate was added as a carbon source at concentrations of 5 g l^−1^. In experiments on complex medium, 5 or 10 g l^−1^ yeast extract were added as indicated. For the defined medium an amino acid mix and vitamins were added from sterile filtered stocks. Amino acids were added from separate stocks at a final concentration of 1 g l^−1^ asparagine * H_2_O (Merck KGaA, Darmstadt, Germany), 1 g l^−1^ monosodium glutamate * H_2_O, 0.3 g l^−1^ arginine, 0.5 g l^−1^ lysine * H_2_O (Merck KGaA, Darmstadt, Germany) and 0.5 g l^−1^ methionine (Merck KGaA, Darmstadt, Germany) in the medium. Asparagine was later replaced by monosodium aspartate * H_2_O (Merck KGaA, Darmstadt, Germany) at concentrations of 1.15 g l^−1^ as indicated. For experiments with TCA cycle intermediates, stocks of 0.88 g l^−1^ succinate, malate (Merck KGaA, Darmstadt, Germany) and oxaloacetate were used.

The vitamin stock solution was adapted according to Pfeifer et al. [35] and the final concentration in the medium was 4.5 mg l^−1^ thiamine hydrochloride, 0.53 mg l^−1^ riboflavin, 6.8 mg l^−1^ calcium D-pantothenate, 7.5 mg l^−1^ nicotinic acid (Merck KGaA, Darmstadt, Germany), 1.75 mg l^−1^ pyridoxine hydrochloride (AppliChem GmbH, Darmstadt, Germany), 0.075 mg l^−1^ biotin (Merck KGaA, Darmstadt, Germany) and 0.05 mg l^−1^ folic acid (Merck KGaA, Darmstadt, Germany). These concentrations accounted for the 1 × concentration and amounts were reduced in some experiments as indicated.

The feed medium contained 100 g l^−1^ acetate. If indicated, 28.8 g l^−1^ monosodium aspartate * H_2_O was added to the feed. Feed medium was pulsed to the cultures to restore an acetate concentration of 4 g l^−1^. Low-pulse volumes potentially accounted for inaccuracies in carbon balances.

Liquid and solid media were supplemented with 50 µg ml^−1^ kanamycin.

### Construction of plasmids and strains

The genes *budA*, *budB* and *budC* from *Enterobacter cloacae* subsp. *dissolvens* were overexpressed for 2,3-butanediol production. Plasmid 445_Ediss previously constructed and transformed into *E. coli* *W* and *E. coli* *W* Δ*ldhA* Δ*adhE* Δ*pta* Δ*frdA* was used in this study [6]. This plasmid was additionally transformed into *E. coli* *W* Δ*ldhA* Δ*adhE* by electroporation.

### Preparation of precultures

All strains and constructs were stored at −  80 °C in 20% (w/v) glycerol. For cultivations, they were streaked onto LB agar plates containing 50 µg ml^−1^ kanamycin and incubated overnight at 37 °C. A single colony was used for inoculation of 500 ml shake flasks with 50 ml LB medium. The preculture was incubated overnight at 37 °C and 230 rpm. Cells were centrifuged at 4800 rpm (2396*g*) for 10 min at room temperature and washed with 25 ml of sterile 0.9% (w/v) NaCl. After resuspension in 5 ml 0.9% (w/v) NaCl, the optical density at 600 nm (OD_600_) was measured and for experiments in shake flasks, the appropriate volume to reach an initial OD_600_ of 0.5 was transferred. For bioreactor experiments, the culture was adapted to growth on acetate in defined medium. To this end, the preculture was sequentially transferred twice into 500 ml shake flasks with 20 ml defined medium containing the 1 × concentration of aspartate and vitamins. The optical density was measured in regular intervals and bioreactors were inoculated with 10 ml of the culture in the exponential growth phase.

### Media and strain screening in shake flasks

Screening of different media compositions and strains was carried out in 500-ml shake flasks containing 20 ml medium as indicated. The flasks were incubated at 30 °C and 230 rpm. Samples were taken every 24 h for OD_600_ and HPLC measurements.

### Cultivations in bioreactors

Bioreactor cultivations were carried out in duplicates in a DASbox^®^ Mini Bioreactor system (Eppendorf AG, Hamburg, Germany) at a working volume of 200 ml and a temperature of 30 °C. The pH was initially set to 7.2 and after initial growth has started, it was changed to 7.0. To avoid precipitations in the continuous cultures, the pH was set to 6.8. The pH was monitored by a pH electrode EasyFerm Plus K8 120 (Hamilton, Reno, NV, USA) and controlled by the addition of 5 M phosphoric acid with a MP8 multi-pump module (Eppendorf AG, Hamburg, Germany). The concentration of dissolved oxygen was monitored by a VisiFerm DO 120 probe (Hamilton, Reno, NV, USA). The agitator speed was kept at 800 rpm and the medium was sparged with 0.2 vvm (2.4 sl h^−1^) air. Gassing rates and stirrer speed were increased to maintain a dissolved oxygen concentration above 30%. Off-gas analysis for O_2_ and CO_2_ was carried out using the gas analyzer module GA4 (Eppendorf AG, Hamburg, Germany).

Samples were taken regularly to measure the optical density at 600 nm and estimate biomass growth. The samples were centrifuged at 14,000 rpm (21,913*g*) for 5 min and the supernatant was used for HPLC analysis of substrate and product concentrations.

For monitoring the acetate concentration during fed-batch cultures, the supernatant was diluted 1:10. Acetate was measured at-line using a Cedex Bio HT Analyzer (Roche, Switzerland).

### Biomass determination

Cell dry weight was determined gravimetrically in duplicates from bioreactor samples at the end of each bioreactor experiment. In short, 4 ml of culture broth was centrifuged in a glass tube at 4800 rpm (2396*g*) and 4 °C for 10 min, washed with 4 ml 0.9% (w/v) NaCl and centrifuged again. The biomass was dried in pre-weighed glass tubes for at least 72 h at 105 °C. The optical density at 600 nm (OD_600_) was measured in a spectrophotometer (Genesys™ 20, Thermo Scientific, Waltham, Massachusetts, USA) against a water blank. The correlation between biomass and OD_600_ was used to estimate the cell dry weight calculation of all other samples.

### HPLC analysis

Organic acids, alcohols and amino acids were determined using an Aminex HPX-87H column (300 × 7.8 mm, Bio-Rad, Hercules/CA, USA) in an Ultimate 3000 system (Thermo Scientific, Waltham/MA, USA). The mobile phase was 4 mM H_2_SO_4_ and the column was operated at 60 °C and a flow of 0.6 ml min^−1^ for 30 min. The injection volume was 10 µl. Detection was performed using a refractive index (Refractomax 520, Thermo Scientific, Waltham/MA, USA) and a DAD detector (Ultimate 3000, Thermo Scientific, Waltham/MA, USA). Chromeleon 7.2.6 Chromatography Data System (Thermo Scientific, Waltham/MA, USA) was used for control, monitoring and evaluation of the analysis.

For the measurement of organic acids and alcohols, 450 µl of culture supernatant were mixed with 50 µl of 40 mM H_2_SO_4_ and centrifuged for 5 min at 14,000 rpm (21,913*g*) at 4 °C. The remaining supernatant was used for further analysis.

For the measurement of amino acids and particularly aspartic acid, 250 µl of culture supernatant was mixed with 50 µl of 1 M sodium nitrite and 10 µl of 12 M HCl. The solution was heated to 45 °C for 90 min and the reaction was stopped by adding 50 µl 2 M NaOH [52]. The derivatized amino acid solution was directly transferred to an HPLC tube and measured at the conditions described above.

Standards were treated like samples and a 5-point calibration was used for quantification.

## Supplementary information

**Additional file 1: supplementary tables**. **Table S1**. Comparison of growth and production in *E. coli *W and *E. coli* W ΔldhA ΔadhE Δpta ΔfrdA (Δ4). **Table S2**. Diol production from acetate and other amino acids in *E. coli *W ΔldhA ΔadhE Δpta ΔfrdA.

## Data Availability

The datasets used and/or analyzed during the current study are available from the corresponding author on reasonable request.
