# Supplementary material for: Microbial upgrading of acetate into 2,3-butanediol and acetoin by E. coli W
Source: Biotechnol Biofuels. 2020 Oct 22;13:177. doi: 10.1186/s13068-020-01816-7 (PMC7584085; doi:10.1186/s13068-020-01816-7)
Supplement: Supplementary file 1 — Additional file 1: supplementary tables. Table S1. Comparison of growth and production in E. coli W and E. coli W ΔldhA ΔadhE Δpta ΔfrdA (Δ4). Table S2. Diol production from acetate and other amino acids in E. coli W ΔldhA ΔadhE Δpta ΔfrdA. [file 13068_2020_1816_MOESM1_ESM.docx]

**Supplementary Tables for**

# Microbial upgrading of acetate into 2,3-butanediol and acetoin by *E. coli* W

Katharina Novak, Regina Kutscha and Stefan Pflügl

**Table S1 – Comparison of growth and production in *E. coli* W and** ***E. coli* W Δ*ldhA* Δ*adhE* Δ*pta* Δ*frdA* (Δ4).** Media contained 5 g l^-1^ acetate and 10 g l^-1^ yeast extract and cultures were inoculated at OD_600_ = 0.5. Means and standard deviations were calculated from biological triplicates.

| **Strain** | ***E. coli* W** | ***E. coli* W Δ4** |
| --- | --- | --- |
| OD_600_ [-] | 5.3 ± 0.1 | 5.8 ± 0.3 |
| Diols [ g l^-1^] | 0.20 ± 0.01 | 0.67 ± 0.03 |

**Table S2 – Diol production from acetate and other amino acids in *E. coli* W Δ*ldhA* Δ*adhE* Δ*pta* Δ*frdA.*** Media contained 5 g l^-1^ acetate and 0.88 g l^-1^ of the corresponding amino acid and cultures were inoculated at OD_600_ = 0.5. Means and standard deviations were calculated from biological triplicates.

| **Amino acid** | **Lysine** | **Methionine** | **Arginine** |
| --- | --- | --- | --- |
| OD_600_ [-] | 2.5 ± 0.6 | 2.2 ± 0.1 | 2.5 ± 0.8 |
| Diols [ g l^-1^] | 0.09 ± 0.03 | 0.06 ± 0.01 | 0.10 ± 0.01 |
